# AI-Enabled Screening for Retinopathy of Prematurity in Low-Resource Settings

**DOI:** 10.1001/jamanetworkopen.2025.7831

**Published:** 2025-04-29

**Authors:** Anthony Ortiz, Susana Patiño, Jehú Torres, Juan Mármol, Carlos Serafin, Rahul Dodhia, Gabriela Saidman, Vanina Schbib, Brenda Peña, Guillermo Monteoliva, María Ana Martinez-Castellanos, William B. Weeks, Juan M. Lavista Ferres

**Affiliations:** 1 Microsoft AI for Good Lab, Redmond, Washington; 2 Business Data Evolution, Mexico City, Mexico; 3 Ophthalmology Department, Hospital Italiano de Buenos Aires, Buenos Aires, Argentina; 4 Asociación Para Evitar la Ceguera en México, Mexico City, Mexico

## Abstract

**Importance:**

Retinopathy of prematurity (ROP) is the leading cause of preventable childhood blindness worldwide. If detected and treated early, ROP-associated blindness is preventable; however, identifying patients who might respond to treatment requires screening over time, which is challenging in low-resource settings where access to pediatric ophthalmologists and pediatric ocular imaging cameras is limited.

**Objective:**

To develop and assess the performance of a machine learning algorithm that uses smartphone-collected videos to perform retinal screening for ROP in low-resource settings.

**Design, Setting, and Participants:**

This diagnostic study used smartphone-obtained videos of fundi in premature neonates with and without ROP in Mexico and Argentina between May 12, 2020, and October 31, 2023. Machine-learning (ML)–driven algorithms were developed to process a video, identify the best frames within the video, and use those frames to determine whether ROP was likely or not. Eligible neonates born with gestational age less than 36 weeks or birth weight less than 1500 g were included on the study.

**Exposures:**

An ML algorithm applied to a smartphone-obtained video.

**Main Outcomes and Measures:**

The ML algorithms’ ability to identify high-quality retinal images and classify those images as indicating ROP or not at the frame and patient levels, measured by accuracy, specificity, and sensitivity, compared with classifications from 3 pediatric ophthalmologists.

**Results:**

A total of 524 videos were collected for 512 neonates with median gestational age of 32 weeks (range, 25-36 weeks) and median birth weight of 1610 g (range, 580-2800 g). The frame selection model identified high-quality retinal images from 397 of 456 videos (87.1%; 95% CI, 84.0%-90.1%) reserved for testing model performance. Across all test videos, 97.4% (95% CI, 96.7%-98.1%) of high-quality retinal images selected by the model contained fundus images. At the frame level, the ROP classifier model had a sensitivity of 76.7% (95% CI, 69.9%-83.5%); at the patient level, the classifier model had a sensitivity of 93.3% (95% CI, 86.4%-100%). At both levels, the model’s sensitivity was higher than that for the panel of pediatric ophthalmologists (frame level: 71.4% [95% CI, 64.1%-78.7%]; patient level: 73.3% [95% CI, 61.0%-85.6%]). Specificity and accuracy were higher for ophthalmologist classification vs the ML model.

**Conclusions and Relevance:**

In this diagnostic study, a process that used smartphone-collected videos of premature neonates’ fundi to determine whether high-quality retinal images were present had high sensitivity to classify such images as indicating or not indicating ROP but lower specificity and accuracy than ophthalmologist assessment. This process costs a fraction of the current process for retinal image collection and classification and could be used to expand access to ROP screening in low-resource settings, with potential to help prevent the most common cause of preventable childhood blindness.

## Key Points

**Question:**

Can machine learning algorithms process and classify smartphone-collected videos of premature neonates’ eye fundi to identify patients exhibiting signs of retinopathy of prematurity (ROP)?

**Findings:**

In this diagnostic study of smartphone-collected videos of fundi in 512 neonates, machine learning algorithms processed and used the videos to classify patients as having or not having ROP with greater sensitivity than a panel of 3 pediatric ophthalmologists. Specificity and accuracy were higher for ophthalmologist classification.

**Meaning:**

The findings suggest that machine learning algorithms using smartphone-collected retinal images could expand access to ROP screening and optimize the scarce pediatric ophthalmologist workforce, particularly in low-resource settings.

## Teaser

This diagnostic study evaluates the performance of machine learning algorithms to identify retinopathy of prematurity using smartphone-collected videos of retinas.

## Introduction

Retinopathy of prematurity (ROP) is a vision-threatening disorder affecting premature infants that results from abnormal retinal vascular development. In later stages, fibrovascular proliferation leads to retinal detachment, visual impairment, and blindness.^1^

Infants at risk for ROP are born prematurely (gestational age, ≤30 weeks) or weigh less than 1500 g at birth.^2^ Because neonatal intensive care has improved, more preterm neonates are living and ROP cases have increased worldwide,^3^ particularly in low- and middle-income countries in Africa^4^ and Latin America,^5^ where no routine screening for ROP occurs. ROP is the leading cause of preventable childhood blindness worldwide,^6^ and between 1990 and 2019, the age-standardized rates of ROP increased, as did the prevalence of childhood and adulthood vision loss due to ROP.^7^ If detected and treated early, ROP-related blindness is preventable.^8^

To enable timely intervention, a screening and monitoring system has been implemented for infants at risk of developing ROP in most high-income countries.^8^ In the US, the initial screening occurs within the first 4 weeks after birth, with regular weekly examinations needed until it becomes evident either that the eyes will not develop a condition requiring treatment or that treatment is required; treatment should be administered within 48 hours of recognition of ROP, as ROP can progress rapidly.^2^ That treatment—a combination of laser application to the areas of concern in the retina and antiangiogenic medications—must be administered by a pediatric ophthalmologist, a rare specialist even in high-resource countries. In the US in March 2022, 4 states and 90% of counties had no pediatric ophthalmologists,^9^ and the US pediatric ophthalmologist workforce shortage is anticipated to worsen.^10^ In Latin America, pediatric ophthalmologists are even more scarce, and access to high-cost indirect ophthalmoscopic devices, required to obtain retinal images for screening, is limited.^5,11^ The increasing incidence of ROP in low-income countries has been attributed to lack of resources, including costly pediatric ocular imaging cameras and access to pediatric ophthalmologists.^3^

While some researchers have applied artificial intelligence (AI) techniques to identify ROP and classify its severity, that body of work is limited because of its reliance on pediatric ocular camera images, which are not generally available in low-resource settings. AI algorithms that have been trained on those images have been used to identify ROP and classify its severity^12^ even in low- and middle-income populations.^13^ However, a systematic review of 27 published algorithms found that all but 3 used pediatric ocular camera images^14^; of these 3, one used poor-quality images from an unspecified source,^15^ another did not identify how retinal images were obtained,^16^ and the third evaluated the quality of images obtained though the i-ROP study,^17^ which used digital wide-field retinal images.^18^

While these AI algorithms might help diagnose and classify retinal images collected with sophisticated technology, we sought to develop an AI-assisted solution that could use images and videos captured with widely accessible smartphone cameras enhanced with low-cost magnifiers. Therefore, we developed a 3-step process that could be used by lightly trained personnel who were not pediatric ophthalmologists to screen premature neonates for ROP using a smartphone camera.

## Methods

### Overview

This diagnostic study was approved by the institutional review board at the Instituto Materno Infantil del Estado de México in Toluca de Lerdo, Mexico. We followed the Transparent Reporting of a Multivariable Prediction Model for Individual Prognosis or Diagnosis for AI (TRIPOD+AI) reporting guideline by adhering to the reporting checklist. Prior to obtaining videos, written informed consent was obtained from each patient’s parent.

We developed a 3-step process to screen premature neonates for ROP. After the patient was prepared and eyes dilated, a video of retinal images was obtained from a smartphone that used the same low-cost magnifier used for indirect ophthalmoscopy. Then, we applied circular Hough transformations to each frame of the video and matched the proposed circles to the expected magnifier parameters, allowing us to detect and crop the retinal image for the frame. The best retinal images for ROP evaluation were selected using a supervised convolutional neural network classifier. If no high-quality images were obtained, the videographer would take another video. The selected best images were analyzed using an image binary classification model to predict a probability of presence of ROP in the frame. Patients classified as ROP positive included those with ROP at stages 1, 2, 3, 4a, or 4b or aggressive ROP, while patients with stage 0, including some patients with preplus disease, were considered ROP negative. The dataset included patients with ROP in zone 1, zone 2 anterior, zone 2 posterior, or zone 3. Each step of the process and the analysis process are further described hereafter.

Neonates who were born with a gestational age of less than 36 weeks or who weighed less than 1500 g at birth were eligible for the study. Participants were recruited from neonatal intensive care units in hospitals in Argentina and Mexico where some of us (G.S., V.S., B.P., G.M.) had admitting privileges.

Patients were enrolled in the study between May 12, 2020, and October 31, 2023. Videos of fundi were obtained before participants reached 28 days of age.

### Video Capture Requirements

**Figure 1.**
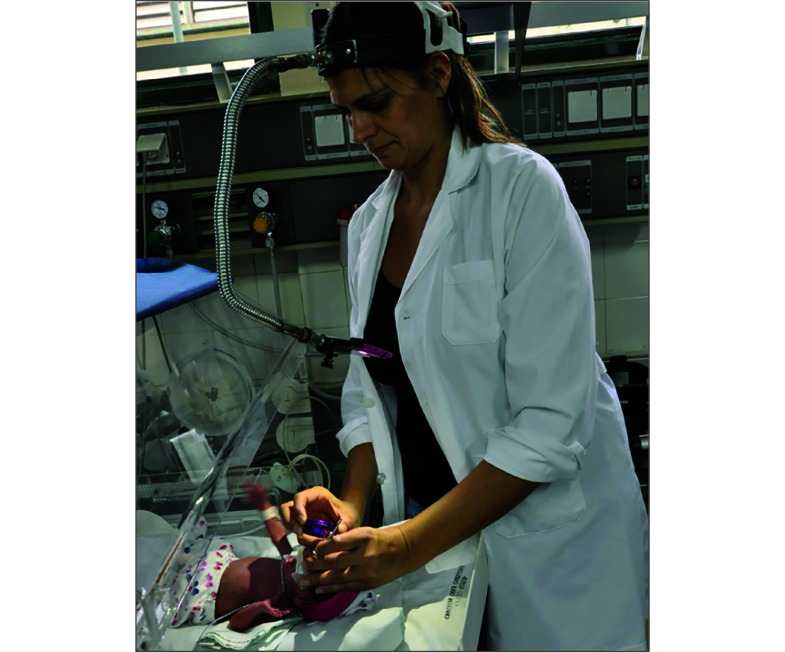
Video Capture of the Retina Using a Low-Cost Magnifier Lens and a Smartphone Mounted to an Adjustable Smartphone Holder

This work was based on videos and images collected using a smartphone mounted on an adjustable headband through a flexible stainless-steel tube and a universal smartphone tripod (Figure 1). The video collection system requires a smartphone with a camera that can record videos with continuous flash lighting and a +20 diopter (D), +28 D, or +40 D condensing aspheric magnifying lens held by the examiner over the dilated eyes of the patient. Our study included videos captured with all aforementioned magnifying lenses from a wide range of cell phones and both mp4 and mov video file formats. The cost of the setup, including the smartphone, is approximately US $1000,^19^ about one-hundredth the cost of a wide-angle contact imaging pediatric ocular imaging system.^20^

### Video Analysis Process

#### Fundus Image Selection

In the first phase, our machine learning algorithm sought to identify the highest-quality retinal images from the smartphone-captured, magnifying glass–enhanced video of the retina. All video frames were extracted from the raw mobile videos at a specific sampling rate of 5 frames, and the individual color video frames were converted to grayscale. Since the magnifiers used for video collection are circular, we used the circular Hough transformation process^21^ to conduct a search of all circles within a frame in a learned radius range. The Hough transformation can be used to determine the parameters of a circle when several points that fall on the perimeter are known, as follows. A circle with radius *R* and center (*a*, *b*) can be described with the parametric equations *x* = *a* + *R* cos(θ) and *y* = *b* + *R* sin(θ). When angle θ sweeps through a 360-degree range, the points *x* and *y* trace the perimeter of a circle. The radius is calculated based on the magnifier size, and the search identifies the true center point of the circle. If no circles with the expected dimensions are found, the frame is disregarded. If multiple circles are detected, the one with parameters that are closest to the expected magnifier dimensions is selected. Using the center coordinate parameters and the radius, the fundus images were cropped from the original color video frame and resized to a fixed 256 ×  256 pixel size.

**Figure 2.**
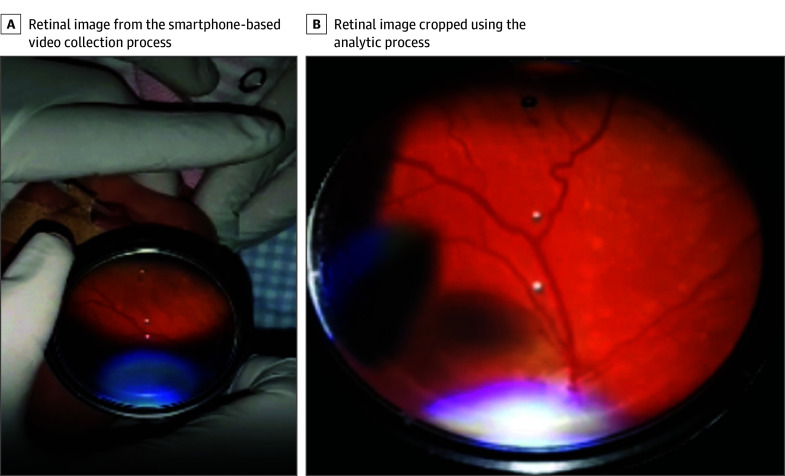
Fundus Images

To ensure that only retinal images were collected, we trained an image classifier that used a residual network^22^ architecture. The output of this phase was a set of fundus images like the one shown in Figure 2B.

#### ROP Classification and Model Calibration

In the second phase, the retinal images from the previous step were sent to an ROP image classifier. We constructed multiple classification models, optimizing them for recall, meaning that we sought to minimize the frequency of false negatives (images that the model identified as indicating that the neonate did not have ROP when, in truth, the image suggested that the neonate did have ROP). The final classifier model used an 18-layer residual network architecture. We trained the image classifier using Adam optimization^23^ with binary weighted cross-entropy loss until there was convergence using a learning rate of 0.0001. During training, we used multiple data augmentation techniques, including image rotation, horizontal and vertical flips, and image jittering.^24^ Since the predicted confidence outputs of the image classifier did not reflect the true probability distribution of the ROP dataset, we used temperature scaling to calibrate output so that it generated calibrated probabilities.^25^

### Model Development, Testing, and Validation

To develop, test, validate, and evaluate our application, we conducted experiments using a dataset of videos collected by pediatric ophthalmologists in Mexico and Argentina. The training dataset included 56 videos that were collected from 28 patients with ROP and 28 individuals without ROP. For model validation, we used 12 additional videos (7 from patients with ROP and 5 from individuals without ROP), and for model testing, we used 456 videos collected by partner pediatric ophthalmologists during the study period. All videos were anonymized. From these videos, 2 independent frame datasets were created: a frame selection dataset and an ROP classification dataset for analysis.

**Figure 3.**
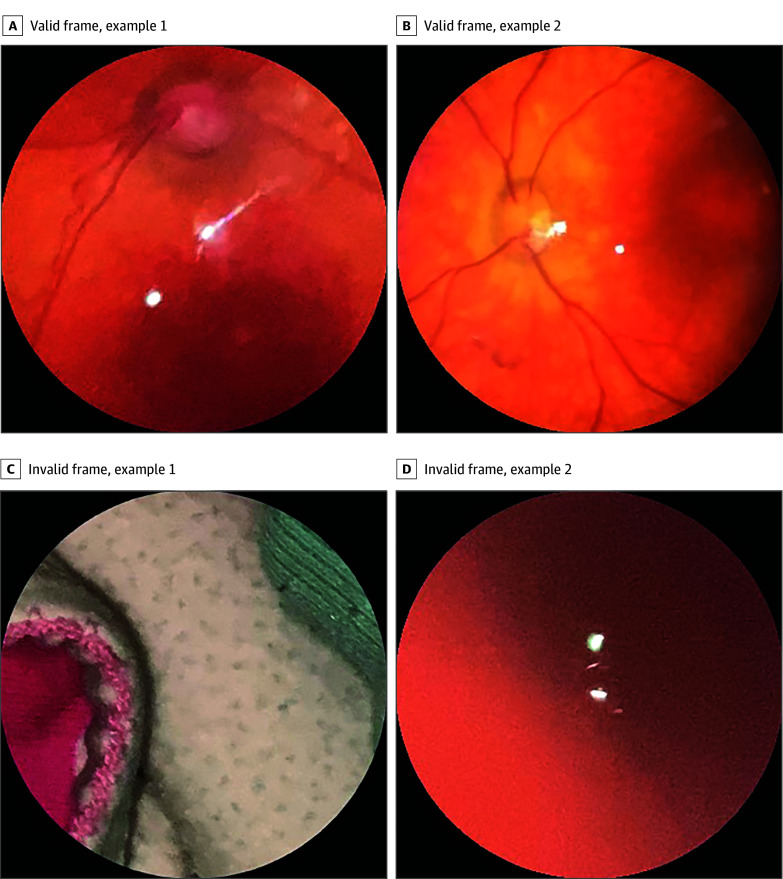
Examples of Valid and Invalid Fundus Video Frames Invalid frames were selected by hard negative mining.

Through hard negative mining, partner pediatric ophthalmologists (G.M., M.A.M.-C.) visually reviewed and iteratively created a frame selection dataset consisting of 370 valid and invalid frames from the training dataset and 100 valid and invalid frames from the validation dataset (examples of valid and invalid frames are provided in Figure 3). Using those frames, we trained a fundus frame selection classifier.

We created an ROP classification dataset consisting of 2227 retinal images. These selected frames were reviewed and labeled by consensus of 3 pediatric ophthalmologists (V.S., B.P., G.M.) as showing or not showing ROP. In all, we used 1664 frames (74.7%; 657 [39.5%] with no ROP, 1007 [60.5%] with ROP) for model training, 272 frames (12.2%; 112 [41.2%] with no ROP, 160 [58.8%] with ROP) for model validation, and 291 frames (13.1%; 122 [41.9%] with no ROP, 169 [58.1%] with ROP) for model testing. Both frame and video splits were created using stratified random sampling. Table 1 provides the characteristics of the different datasets used and a summary of how they were used and how their use was evaluated.

**Table 1.** Dataset Characteristics, Use, and Evaluation

	Dataset			Use and evaluation
	Training	Model validation	Model testing	
Videos, No.	56 (28 [50.0%] with ROP, 28 [50.0%] without ROP)	12 (7 [58.3%] with ROP, 5 [41.7%] without ROP)	456 Collected by clinicians screening for ROP	All 456 videos in the model-testing dataset were used to test the fundus frame selection model; 50 randomly selected videos from the model-testing dataset were used to identify 148 test frames that were used to compare the accuracy, specificity, and sensitivity of the model with that of pediatric ophthalmologists’ predictions at the frame and patient levels
Frame selection dataset	370 Valid and invalid fundus frames	100 Valid and invalid fundus frames	NA	Used for fundus frame selection classification training
ROP classification dataset	1664 Valid frames (1007 [60.5%] labeled as ROP, 657 [39.5%] as non-ROP)	272 Valid frames (160 [58.8%] labeled as ROP, 112 [41.2%] as non-ROP)	291 Valid frames (169 [58.1%] labeled as ROP, 122 [41.9%] as non-ROP)	Used to determine whether the frame selection model could obtain high-quality retinal images, calculate the precision of the frame selection model, and evaluate the model at the frame level, using the pediatric ophthalmologists’ determinations as the gold standard

Abbreviations: NA, not applicable; ROP, retinopathy of prematurity.

#### Model Performance Evaluation

The goal of the frame selection model was to obtain a useful set of retinal images from each video. To test the model’s ability to do so, we applied the frame-selection framework to all 456 videos in the model testing dataset. A prediction was considered correct if visual inspection of the selected frames included clear fundus images. We calculated the model’s precision as the proportion of all selected frames that included a clear retinal image.

We evaluated the ROP classifier across 2 scenarios. First, we used the model testing dataset to evaluate the classifier at the video and patient levels, wherein a consensus of 3 pediatric ophthalmologists who determined whether ROP was present was the gold standard.

Second, we evaluated the classifier at the patient and frame levels in the clinic. For 50 randomly chosen videos, we ran our frame selection process to identify 148 test frames (having selected the 3 retinal images with the highest quality ranking from 48 videos and the 2 retinal images with the highest quality ranking from 2 videos); these frames were then randomized and shown to 3 pediatric ophthalmologists who are ROP experts and who classified each frame as ROP positive, ROP negative, or impossible to determine. If all 3 marked a frame as impossible to determine, it was disregarded. Of note, the patients had already been diagnosed by a pediatric ophthalmologist based on the entire videos, but now the diagnosis had to be made from a single frame. If any of the 3 selected frames was classified as positive for ROP, the patient was considered to have ROP. Of note, the consensus from the 3 pediatric ophthalmologists from the entire video analysis was still the gold standard. We measured the accuracy (the proportion of correct predictions [ROP and non-ROP] among the total cases evaluated by the model), the specificity (the proportion of patients without ROP who were correctly determined not to have ROP), and the sensitivity (the proportion of patients with ROP who were correctly determined to have ROP) of each pediatric ophthalmologist, of the consensus of the ophthalmologists, and of the model. Since the purpose of our work was to screen patients for further evaluation by scarce specialists, results were always validated by a pediatric ophthalmologist. Because it is worse from a patient care perspective to miss an ROP case than it is to raise a concern about ROP when there is none, we optimized our ROP model for sensitivity by adding a higher penalty in the loss function for false-negative predictions using weighted binary cross-entropy loss during training.

### Statistical Analysis

We used the normal approximation interval method to compute the 95% CI for each accuracy, specificity, and sensitivity metric. We assumed that the predictions followed a normal distribution to compute the 95% CI for the mean on a single training-test split under the central limit theorem.^26^ Data were analyzed using SciPy, version 1.14.2 (Python Software Foundation).

## Results

A total of 524 videos were collected for 512 premature neonates enrolled in the study, with median gestational age of 32 weeks (range, 25-36 weeks) and median birth weight of 1610 g (range, 580-2800 g). The median weight of neonates was 1735 g (range, 670-3000 g) after 28 days of age. Altogether, 456 videos (89.1%) were held out for testing. A total of 361 neonates (70.5%) did not have ROP, while 151 (29.5%) were ROP positive. Of the neonates with ROP, 64 (42.3%) had stage 1; 45 (29.8%), stage 2; 22 (14.6%), aggressive; 14 (9.3%), stage 3; and 6 (4.0%), stage 4a or 4b. Our frame selection model was able to identify high-quality retinal images, with the number of images identified being a function of the sampling rate. When sampling from every 5 frames, the process obtained high-quality retinal images from 376 test videos (82.5%; 95% CI, 79.0%-86.0%); when sampling from every other frame, high-quality retinal images were obtained from 397 (87.1%; 95% CI, 84.0%-90.1%). Across all test videos, we found a precision of 97.4% (95% CI, 96.7%-98.1%) at the frame level, and the area under the receiver operating characteristic curve was 0.96 (95% CI, 0.95-0.97).

Next, we evaluated the model’s and individual experts’ performance on the 148 random individual frames from 50 different patients. When evaluating the performance of the ROP classifier, at the frame level, the model had a sensitivity of 76.7% (95% CI, 69.9%-83.5%), meaning that 76.7% of the time, our model predicted ROP in frames from videos that had been determined by ophthalmologists to indicate ROP (Table 2). The model’s sensitivity was considerably higher than that for any of the 3 pediatric ophthalmologists tested and for the consensus (71.4%; 95% CI, 64.1%-78.7%).

**Table 2.** Performance of Individual Pediatric Ophthalmologists, the Consensus Among Them, and the Model at the Frame and Patient Levels

Classifier	Metric (95% CI), %					
	Frame level			Patient level		
	Accuracy	Specificity	Sensitivity	Accuracy	Specificity	Sensitivity
Ophthalmologist						
1	76.6 (69.8-83.4)	78.6 (72.0-85.2)	64.3 (56.6-72.0)	71.1 (58.5-83.7)	72.4 (60.0-84.8)	73.3 (61.0-85.6)
2	75.8 (68.9-82.7)	78.6 (72.0-85.2)	64.3 (56.6-72.0)	73.3 (61.0-85.6)	75.9 (64.0-87.8)	66.7 (53.6-79.8)
3	77.1 (70.3-83.9)	74.2 (60.5-85.2)	65.9 (58.3-73.5)	72.9 (60.5-85.2)	75.9 (64.0-87.8)	68.8 (56.0-81.6)
Consensus	78.0 (71.3-84.7)	81.9 (75.7-88.1)	71.4 (64.1-78.7)	76.7 (65.0-88.4)	75.9 (64.0-87.8)	73.3 (61.0-85.6)
Machine learning model	64.9 (57.2-72.6)	64.3 (56.6-72.0)	76.7 (69.9-83.5)	66.7 (53.6-79.8)	55.2 (41.4-69.0)	93.3 (86.4-100)

At the patient level, although our model was not as accurate or as specific as the pediatric ophthalmologists, the model outperformed them by a large margin in sensitivity, with a value of 93.3% (95% CI, 86.4%-100%) compared with 73.3% (95% CI, 61.0%-85.6%) for the consensus of ophthalmologists (Table 2). Note that for the patient-level analysis, if any of the 3 frames evaluated was considered to show ROP presence, the diagnosis for the patient was assumed as ROP positive, explaining the discrepancy in performance between frame level and patient level.

## Discussion

In this diagnostic study, we developed a process of using smartphone-obtained videos of retinas to identify frames with good resolution and then subjected such frames to a machine learning algorithm to identify frames likely to suggest ROP. Our process was less specific or accurate than a panel of 3 pediatric ophthalmologists; however, our process was more sensitive than that panel. Of note, in the analysis of a single frame, the pediatric ophthalmologist determined the diagnosis with limited information, and it is possible that some frames did not show the affected ROP zone. Screening at the patient level vs frame level is recommended. Given that missing a patient with ROP was worse than identifying only patients with ROP, the results suggest that our low-cost process can be used in low-resource settings to improve the efficiency and effectiveness of pediatric ophthalmologists by steering only patients who are likely to have ROP to them, thereby essentially multiplying this scarce workforce by reducing the time they spend examining patients without ROP. Furthermore, the video recording process required minimal training, which would improve access to screening.

Importantly, the immediate upload and evaluation of videos allowed for quality control and reduced patient exposure to anesthesia; if there were an inadequate number of frames for analysis, another video could be obtained while the patient was prepared. Furthermore, because ROP can develop over time, our approach allows for easy monitoring by conducting screening over time while the patient is susceptible.

### Limitations

Our study has several limitations. First, we used a limited number of videos and labeled fundus images to create our analytic process, and these were collected in Latin America. Further work is required to assure generalizability to other populations. Second, we compared the accuracy, sensitivity, and specificity of our model with those of only 3 pediatric ophthalmologists. While we randomized the images, conducted analyses at the patient and image levels, and found that performance of the ophthalmologists was similar, comparisons with findings from a larger number of specialists is warranted. Smartphone imaging, even with magnification, may not capture the same level of detail as pediatric ocular camera imaging. The reliance on images selected from videos introduces variability in the areas captured and might miss affected areas. In addition, some of the collected videos were not usable because of missing fundus frames of good quality. The frame selection process should be integrated with the collection to request a new video collection at the point of care.

## Conclusions

In this diagnostic study, a process that used smartphone-collected videos of premature neonates’ fundi to determine whether high-quality retinal images were present had high sensitivity to classify such images as indicating or not indicating ROP but had lower specificity and accuracy than pediatric ophthalmologist classification. This usable process may substantially increase screening for ROP in low-resource settings. As screening is one of the major limitations of receiving ROP treatment, by offloading the process to a less scarce physician type with less training, our process could liberate pediatric ophthalmologists to focus more on activities that only they are qualified to do, such as diagnosis and treatment, and less on conducting screening.

## Notes

Supplement.
Data Sharing Statement
