# Supplementary material for: AI-Enabled Screening for Retinopathy of Prematurity in Low-Resource Settings
Source: JAMA Netw Open. 2025 Apr 29;8(4):e257831. doi: 10.1001/jamanetworkopen.2025.7831 (PMC12042057; doi:10.1001/jamanetworkopen.2025.7831)
Supplement: Supplement. — Data Sharing Statement [file jamanetwopen-e257831-s001.pdf]

Data Sharing Statement

Ortiz. AI-Enabled Screening for Retinopathy of Prematurity in Low-Resource Settings. *JAMA Netw Open*. Published April 29, 2025. doi:10.1001/jamanetworkopen.2025.7831

Data
Data available: Yes
Data types: Deidentified participant data
How to access data: Request data to: anthony.ortiz@microsoft.com
When available: With publication

Supporting Documents
Document types: None

Additional Information
Who can access the data: researchers whose proposed use of the data has been approved
Types of analyses: For academic purposes
Mechanisms of data availability: without investigator support
